# Study of the influence of meteorological factors on HFMD and prediction based on the LSTM algorithm in Fuzhou, China

**DOI:** 10.1186/s12879-023-08184-1

**Published:** 2023-05-05

**Authors:** Hansong Zhu, Si Chen, Rui Liang, Yulin Feng, Aynur Joldosh, Zhonghang Xie, Guangmin Chen, Lingfang Li, Kaizhi Chen, Yuanyuan Fang, Jianming Ou

**Affiliations:** 1 Fujian Provincial Center for Disease Control and Prevention, Fujian Provincial Key Laboratory of Zoonosis Research, The Practice Base On the School of Public Health Fujian Medical University, Fuzhou, Fujian 350012 China; 2 Fujian Climate Center, Fuzhou, 350028 Fujian China; 3 Department of Nutrition, The First Affiliated Hospital of Zhengzhou University, Zhengzhou, 450052 Henan China; 4 School of Public Health, Fujian Medical University, Fuzhou, 350108 Fujian China; 5 School of Public Health, Xiamen University, Xiamen, 361005 Fujian China; 6 College of Computer and Data Science, Fuzhou University, Fuzhou, 350108 Fujian China; 7 Department of Pediatric Surgery, Fujian Children’s Hospital, Fuzhou, 350001 Fujian China

**Keywords:** Meteorological, Relative humidity, Air temperature, HFMD, LSTM, DLNM

## Abstract

**Background:**

This study adopted complete meteorological indicators, including eight items, to explore their impact on hand, foot, and mouth disease (HFMD) in Fuzhou, and predict the incidence of HFMD through the long short-term memory (LSTM) neural network algorithm of artificial intelligence.

**Method:**

A distributed lag nonlinear model (DLNM) was used to analyse the influence of meteorological factors on HFMD in Fuzhou from 2010 to 2021. Then, the numbers of HFMD cases in 2019, 2020 and 2021 were predicted using the LSTM model through multifactor single-step and multistep rolling methods. The root mean square error (RMSE), mean absolute error (MAE), mean absolute percentage error (MAPE) and symmetric mean absolute percentage error (SMAPE) were used to evaluate the accuracy of the model predictions.

**Results:**

Overall, the effect of daily precipitation on HFMD was not significant. Low (4 hPa) and high (≥ 21 hPa) daily air pressure difference (PRSD) and low (< 7 °C) and high (> 12 °C) daily air temperature difference (TEMD) were risk factors for HFMD. The RMSE, MAE, MAPE and SMAPE of using the weekly multifactor data to predict the cases of HFMD on the following day, from 2019 to 2021, were lower than those of using the daily multifactor data to predict the cases of HFMD on the following day. In particular, the RMSE, MAE, MAPE and SMAPE of using weekly multifactor data to predict the following week's daily average cases of HFMD were much lower, and similar results were also found in urban and rural areas, which indicating that this approach was more accurate.

**Conclusion:**

This study’s LSTM models combined with meteorological factors (excluding PRE) can be used to accurately predict HFMD in Fuzhou, especially the method of predicting the daily average cases of HFMD in the following week using weekly multifactor data.

## Background

Hand, foot, and mouth disease (HFMD) is a common infectious disease in children caused by enterovirus infection. Its symptoms are mainly oral pain, anorexia, fever, and minor herpes or ulcers in the hands, feet, mouth, and other body parts. It can lead to fatal complications in severe cases, such as myocarditis, pulmonary oedema, and aseptic meningoencephalitis [1, 2].

HFMD can be transmitted through contact with respiratory secretions, droplets, and pollutants from infected individuals or through the faecal-oral route, which can easily cause school aggregation events, thus affecting children's everyday life and learning. HFMD has led to many outbreaks worldwide and has become a public health problem in Asia [3]. In recent years, the reported incidence of HFMD ranks second only to viral hepatitis among infectious diseases classified under the Infectious Disease Control and Prevention Act in Fujian Province, China, with a substantial significant social impact that has attracted considerable attention from relevant departments.

Meteorological factors have been recognized as risk factors associated with HFMD epidemics [4-7]. Researchers from various countries and regions have studied the impact of climate on HFMD, including air temperature, sunshine, relative humidity, wind speed, and precipitation. The findings, however, have not been entirely consistent. For instance, several studies have shown that the incidence of HFMD significantly increases as the air temperature increases. Nevertheless, in some studies that concluded that HFMD was not significantly affected by air temperature, the air temperature range that affects HFMD was not exactly the same [7-14]. It has been reported that the impact of sunshine on HFMD increases with increasing sunshine intensity. However, another study showed a negative correlation between sunshine duration and the risk of HFMD infection [15-17]. The reasons for these differences include different analysis model schemes [e.g., generalized linear model (GLM), spatiotemporal zero-inflated negative binomial (ZINB) models, generalized additive mixed model (GAMM), distributed lag nonlinear models (DLNMs)], data types (e.g., daily data, weekly data and monthly data), and region-specific characteristics (e.g., socioeconomic factors, living environment, etc.) that may change the impact of meteorological factors on HFMD [7-18].

**Fig. 1:**
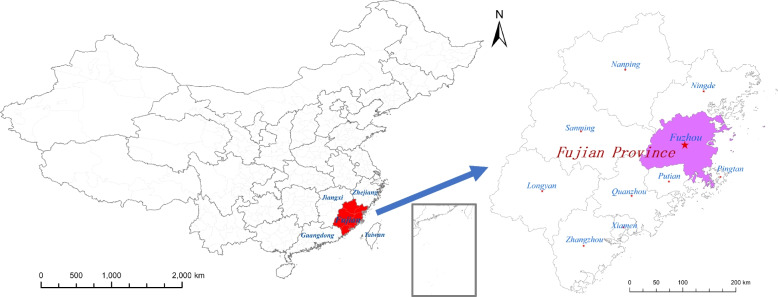
The geographical location of Fuzhou city

Fuzhou is the capital of Fujian Province in China and the province’s political, economic, and cultural centre (Fig. 1). It is an important city along the southeast coast of China and the gateway of the maritime Silk Road. The meteorological characteristics of Fuzhou are characterized by high wind, air pressure, and relative humidity. Fuzhou has the greatest incidence of HFMD among cities in Fujian Province. There has been no report on the influence of meteorological factors on HFMD and incidence prediction in Fuzhou. Therefore, considering the importance of Fuzhou and its representativeness in Fujian, it is necessary to understand the specific regional impact of meteorological factors on HFMD in Fuzhou.

The advantages of DLNM include that it can solve the nonlinear time-delay correlation problems such as exposure-delay-response through the cross-basis function, and it can also automatically deal with the regression functions linear model (lm), glm and gam. Zero-inflated model cannot examine how or which covariates significantly affect the non-occurrence Zero-inflated regions [12]. In this study, DLNMs were proposed to analyse the relationship between the daily values of HFMD and meteorological factors in Fuzhou for 12 years from 2010 to 2021. There were eight meteorological indicators used in this study, including common indicators such as air temperature, relative humidity, precipitation, and sunshine, and other indicators that researchers do not commonly use. At present, there has been no research report on the impact of air pressure differences and air temperature differences on HFMD.

Compared with traditional machine learning methods, long short-term memory (LSTM) produces better results in the deep learning model [19-24]. Previous reports included comparisons between LSTM and other prediction methods, as well as between single-factor and multifactor LSTM predictions. To the best of our knowledge, no studies have compared the prediction accuracy for HFMD using different meteorological multifactor LSTM methods. In this study, the cases of HFMD were combined with meteorological variables, and the cases of HFMD were predicted using the LSTM model through multifactor single-step and multistep rolling methods, and the prediction effect was evaluated. The purpose was to provide a basis and technical support for constructing an HFMD prediction and early warning system in Fuzhou city and Fujian Province, and to help relevant departments detect and respond to possible HFMD outbreaks in advance.

## Materials and methods

### Data sources

The HFMD and population data of Fuzhou from January 1, 2010, to December 31, 2021, were derived from the China Disease Prevention and Control Information System, and the daily meteorological data were derived from the meteorological data network of the China Meteorological Administration (http://data.cma.cn). The missing data were proofread and completed by the Fujian Climate Center. The population with HFMD was stratified by sex (male and female), age (0 ~ 3 years, 4 ~ 6 years, and ≥ 7 years) and area (urban and rural), of which the age-stratified population was divided according to the epidemiological characteristics of HFMD in Fuzhou. The meteorological factors in this study included 8 indicators: air pressure (PRS, hPa), air pressure difference (PRSD, hPa), relative humidity (RHU, %), precipitation (PRE, mm), air temperature (TEM, °C), air temperature difference (TEMD, °C), wind speed (WIN, m/s), and sunshine duration (SSD, h). PRS, RHU, TEM and WIN were measured as daily averages, PRE was measured as the daily cumulative precipitation, SSD was measured as the number of sunshine hours in one day, PRSD was defined as the difference between the maximum and minimum values of daily air pressure, and TEMD was defined as the difference between the highest and lowest values of daily air temperature. The number of lag days in this study was defined as the number of days delayed by the date of HFMD onset compared to the statistical date of the corresponding meteorological factors.

### Statistical analysis of the data

The regional map of Fig. 1 was drawn using ArcGIS 10.2 software (ESRI, Redlands, CA, USA).

R 4.1.0 software (R Foundation for Statistical Computing, Vienna, Austria) was used to analyse the daily HFMD and meteorological data. First, a simple analysis of the HFMD and meteorological factors was conducted, and the time series for the variables were plotted. Then, a Spearman correlation analysis and correlation coefficient significance test map between the meteorological indicators and HFMD were generated, and differences with *P* < 0.05 were considered statistically significant. Finally, a DLNM was used to analyse the influence of meteorological factors on HFMD.

The DLNM incorporates both nonlinear dependency and delay effects, with the essential goal of adding a lag dimension to the exposure–response relationship through a cross-basis function, thereby describing the variation distribution of its effects in both the independent and lagging dimensions [25]. A cross-base matrix for daily meteorological and HFMD data was established, and the quasi-Poisson connection function was used for estimation. After controlling for the effects of day of the week, seasonality and long-term trends [26, 27], the relationship between meteorological factors and HFMD was fitted using the DLNM. The basic model is as follows:

1 $$log[E(Yt)] = \alpha +\beta ixi + NS(Zj, df)+Dow$$

*Yt* is the t-day cases of HFMD, *α* is the constant term, *xi* is the influencing factor, *βi* is the coefficient, *Zj* is the potential confounding factor, *Dow* is the dummy variable for the effect of the day of the week, *df* is the degrees of freedom, and *NS* (⋯) is a natural spline function. Lag days and df are determined by the Akaike information criterion (AIC) minimum criterion, which ultimately determined that the df of meteorological factors in this study were all 3. Accounting for the epidemic characteristics, incubation period, and pretest results of HFMD, the maximum lag days were determined to be 14 days, and the cumulative effects of meteorological factors on the risk of HFMD in each population were measured with lags of 3 d, 7 d and 14 d. The average of each meteorological factor was used as a reference value.

Python 3.8.13 software (Python Software Foundation, Delaware, USA) and Tensorflow 2.8.0 software (Google Brain Team, Mountain View, CA, USA) were used to predict the daily and weekly cases of HFMD through LSTM combined with meteorological factors, and the results were plotted.

LSTM is an artificial intelligence deep learning algorithm that is suitable for time series data analysis. Its key feature is the ability to connect the network model in front of and behind neurons so that the network can process the time series data from both directions. The neurons change their state information with the previous data flow, process the current input data according to the current state and output the results. This structure gives neurons a certain memory ability. LSTM has a well-designed structure called a gate to remove or add information to the neuron state to avoid the problem of long-term dependence and retain the long-term information in the sequence. Gates provide a means for information to be passed selectively. LSTM has three gates, a forgetting gate, an input gate and an output gate, to protect and control the state of neurons.

**Fig. 2:**
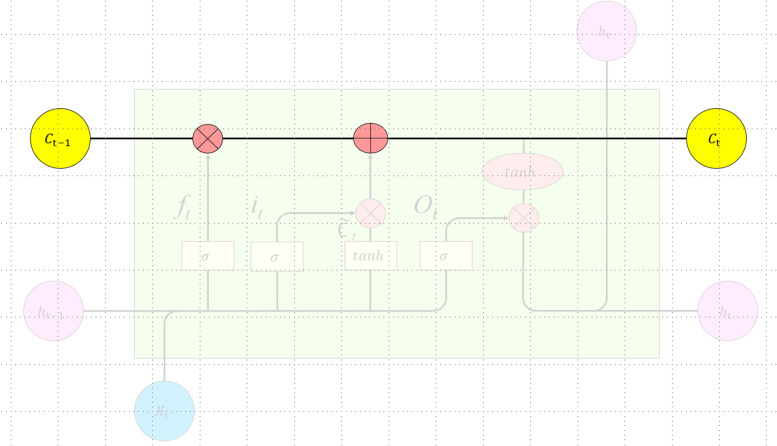
LSTM core idea structure diagram

The core idea of LSTM is shown in Fig. 2.

The first step is to decide what information to discard from the neuron state, which is done through the sigmoid layer of the "forgetting gate". *h*_t−1_ represents the output of the previous neuron state, *X*_t_ represents the input of the current neuron state, and *σ* represents the sigmoid function. The sigmoid layer outputs a numeric value between 0 and 1, denoting how much of each part can pass through, with 0 representing complete discard and 1 representing complete retention. The expression is as follows:

2 $${f}_{t} = \sigma \left({W}_{f} \left[{h}_{t-1}, {x}_{t}\right]+{b}_{f}\right)$$

The second step is to determine what kind of new information is stored in the neuron state. There are two parts here: first, the sigmoid layer of the "input gate" determines which value will be updated; then, the tanh layer creates a new candidate value vector _*t*_ (a value between -1 and 1) that is added to the state and multiplied by the value of the sigmoid function, updating the old neuron state; *C*_*t*-1_ is updated to *C*_*t*_, and finally, the output determines the part to output.

The expression is as follows:

3 $$i_t=\sigma\left(W_i\cdot\left[h_{t-1},x_t\right]+b_i\right)$$

4 $${\widetilde C}_t=\tanh\left(W_c\cdot\left[h_{t-1},x_t\right]+b_c\right.$$

5 $$C_t=f_t\cdot C_{t-1}+i_t\cdot{\widetilde C}_t$$

Finally, the output must be determined by the "output gate". First the sigmoid layer is run to determine which part of the neuron state to output; then, the neuron state is processed by tanh (given a value between -1 and 1) and multiplied by the output of the sigmoid gate, and finally, the determined part is output. The expression is as follows:

6 $$o_t=\sigma\left(W_o\cdot\left[h_{t-1},\;x_t\right]+b_o\right)$$

7 $$h_t=o_t\cdot\tanh(C_t)$$

The root mean square error (RMSE), mean absolute error (MAE), mean absolute percentage error (MAPE) and symmetric mean absolute percentage error (SMAPE) were used to quantify the accuracy of the model's predictions, and the smaller the value was, the higher the prediction accuracy and the higher the confidence [28-31].

The RMSE calculation formula is as follows:

8 $$\mathrm{RMSE}=\sqrt{{\frac{1}{\mathrm{n}}\sum\nolimits_{\mathrm{i=1}}^{\mathrm{n}}}\left({\mathrm{P}}_{\mathrm{i}}-{\mathrm{X}}_{\mathrm{i}}\right)^2}$$

The MAE calculation formula is as follows:

9 $$\mathrm{MAE}=\frac{\sum_{\mathrm i=1}^{\mathrm n}\left|{\mathrm X}_{\mathrm i}-{\widehat{\mathrm X}}_{\mathrm i}\right|}{\mathrm n}$$

The MAPE calculation formula is as follows:

10 $$\mathrm{MAPE}=\frac{100\%}{n}\sum\nolimits_{i=1}^{n}\left|\frac{{P}_{i}-{X}_{i}}{{P}_{i}}\right|$$

The SMAPE calculation formula is as follows:

11 $$\mathrm{SMAPE}=\frac{100\mathrm{\%}}{n}\sum\nolimits_{i=1}^{n}\frac{\left|{P}_{i}-{X}_{i}\right|}{\left|\frac{\left|{P}_{i}\right|+\left|{X}_{i}\right|}{2}\right|}$$

In the above formulas, *Pi* is the observed daily incidence of influenza cases on the *i* day, and *Xi* is the predicted daily incidence of influenza cases on the *i* day where *i* = 1…, *n* [30].

**Fig. 3:**
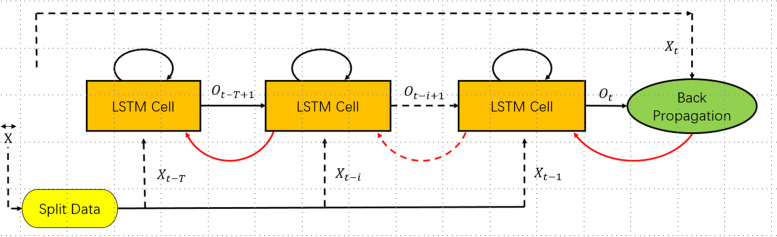
A brief analysis of the LSTM operation process in this study

In this study, we designed a prediction algorithm based on LSTM to capture the temporal relationship in the sequence. The network model was trained with historical data until it converged. The historical time series data were multifactorial, including time, climate data and HFMD data. After coding, LSTM was input to capture the timing relationship, and then the fully connected layer was entered after coding and splicing to output the timing prediction. A brief description of the operation is shown in Fig. 3.

First, the meteorological and HFMD data from 2010 to 2018 were trained and modelled to predict the cases of HFMD in 2019. Then, the data from 2010 to 2019 were trained and modelled to predict the cases of HFMD in 2020. Finally, the data from 2010 to 2020 were trained and modelled to predict the cases of HFMD in 2021. The prediction was realized by single-step and multistep rolling. Three schemes were adopted in this study. The first method was to input the multifactor value of 1 day to predict the cases of HFMD on the next day. The second method was to input the multifactor value of 7 days to predict the cases of HFMD on the next day. The third method was to input the multifactor value of 7 days to predict the daily average cases of HFMD in the next 7 days. These prediction methods required continuous rolling.

## Results

### Descriptive statistics

In total, 161,477 HFMD cases were reported in Fuzhou over the study period, with an incidence rate of 187.42/100,000 people and 16 deaths. The incidence rates (1/100000) among males and females were 222.97 and 143.70, respectively. The incidence rates (1/100000) among children aged 0 ~ 3 years, children aged 4 ~ 6 years and children aged ≥ 7 years were 3321.40, 1030.99 and 7.19, respectively.

Table 1 shows significant differences between the sex, age, and area groups of the HFMD-affected population (*P* < 0.001). Table 2 reports the descriptive statistics for the daily cases of HFMD and meteorological variables.

**Table 1:** Stratified HFMD characteristics of populations based on daily cases

Variables	Sex		Age (years)			Area	
	Males	Females	0 ~ 3	4 ~ 6	≥ 7	Urban	Rural
Cases	99,424	62,053	130,261	25,535	5681	51,234	110,243
Constituent ratio(%)	61.57	38.43	80.67	15.81	3.52	31.73	68.27
t/F	80.29		2664.00			74.86	
p	0.00		0.00			0.00

**Table 2:** Descriptive statistics for the daily cases of HFMD and meteorological variables

Variables	Min	P25	M50	P75	Max	Mean ± SD
HFMD(cases)	0.00	9.00	23.00	49.00	319.00	36.84 ± 40.53
Sex						
Male	0.00	6.00	14.00	31.00	217.00	22.68 ± 25.27
Female	0.00	4.00	9.00	19.00	122.00	14.16 ± 15.75
Ages(years)						
0 ~ 3	0.00	7.00	19.00	41.00	281.00	29.72 ± 32.93
4 ~ 6	0.00	1.00	3.00	7.00	84.00	5.83 ± 7.95
≥ 7	0.00	0.00	1.00	2.00	16.00	1.30 ± 1.89
Area						
Urban	0.00	3.00	7.00	16.00	105.00	11.69 ± 12.94
Rural	0.00	6.00	16.00	33.00	229.00	25.15 ± 28.35
PRS(hPa)	978.50	998.90	1005.00	1011.00	1026.20	1005.00 ± 7.49
PRSD(hPa)	1.40	3.60	4.50	5.60	24.50	4.80 ± 1.75
RHU(%)	27.00	65.00	73.00	82.80	100.00	73.45 ± 12.53
PRE(mm)	0.00	0.00	0.00	2.10	244.40	4.22 ± 12.37
TEM(°C)	2.30	15.00	21.40	27.20	33.40	20.83 ± 7.02
TEMD(°C)	0.60	4.80	7.50	9.70	17.70	7.41 ± 3.25
WIN(m/s)	0.55	1.70	2.10	2.60	9.10	2.20 ± 0.74
SSD(h)	0.00	0.00	3.40	7.80	12.40	4.10 ± 3.90

Min stands for minimum value, Max stands for maximum value, SD stands for standard deviation, P25 stands for 25th percentile, P50 stands for 50th percentile and P75 stands for 75th percentile

**Fig. 4:**
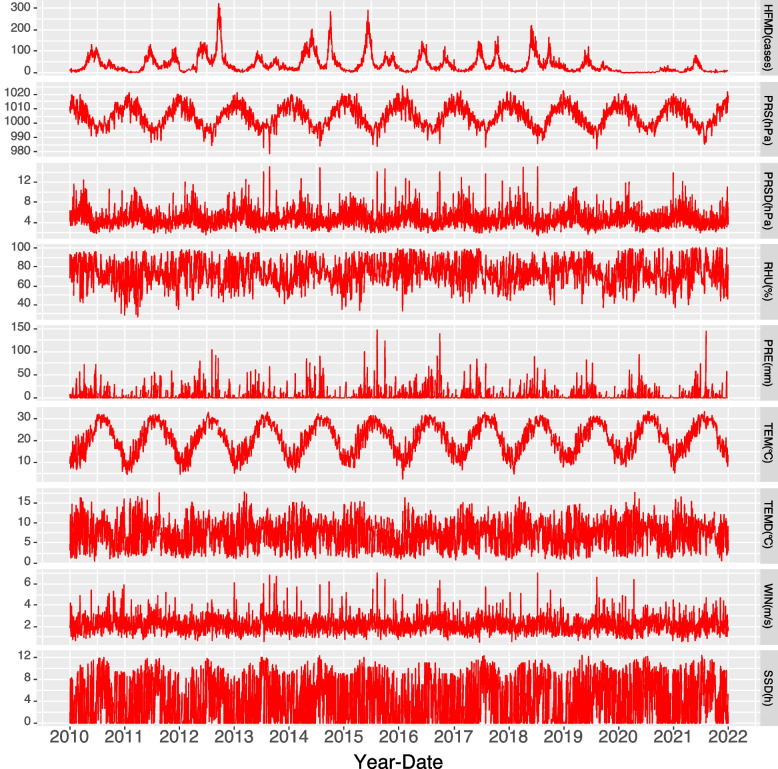
Time series of HFMD cases and meteorological factors. (Note: Some extremely high meteorological factor values were not included in this map)

Figure 4 shows the time series of HFMD and meteorological factors, with a specific seasonal periodicity that shows consistency in their fluctuations, thus indicating a correlation and lag between HFMD and meteorological factors.

### Correlation analysis

**Fig. 5:**
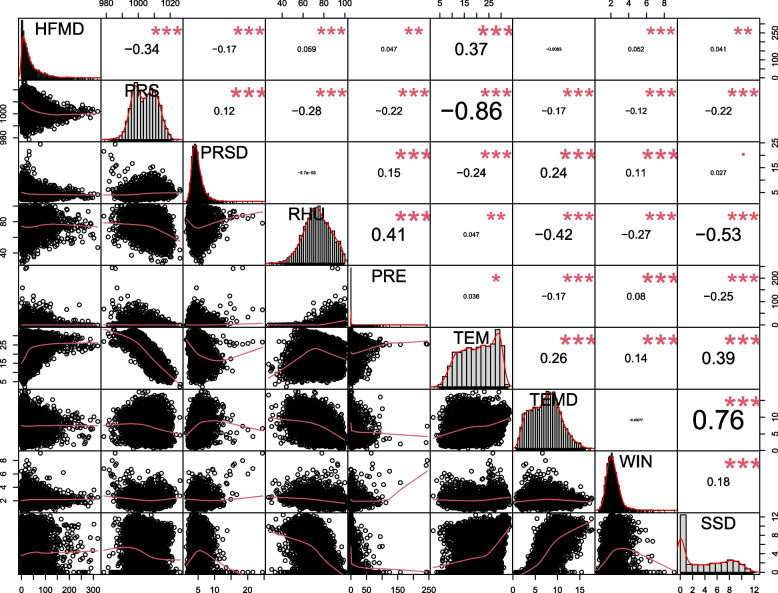
Spearman grade correlation analysis between HFMD and meteorological factors. (Note: '***':* P* < 0.00, '**':* P* < 0.01, '*':* P* < 0.05, '.':* P* < 0.10)

The correlation analysis demonstrated a curved correlation between most meteorological factors and HFMD and between the meteorological factors (*P* < 0.05). RHU, PRE, TEM, WIN, and SSD were significantly positively correlated with HFMD (*r* > 0, *P* < 0.01), while PRS and PRSD were significantly negatively correlated with HFMD (*r* < 0, *P* < 0.01). Among them, TEM, PRS, and PRSD had the most significant relationship with HFMD, while the relationship between TEMD and HFMD was not noticeable. PRS, PRE, and TEM were significantly correlated with other meteorological factors (*P* < 0.05). The detailed correlation between HFMD and meteorological factors is presented in Fig. 5.

### DLNM analysis

The risk effect of PRS on HFMD increased gradually in waves with the increase in PRS. Medium PRS (993–1005 hPa) and high PRS (> 1015 hPa) were risk factors for HFMD. The cumulative effect increased with the increase in lag days, and the correlation peaks were 998 hPa (lag 14 d, RR: 1.36, 95% CI: 1.24–1.48) and 1026 hPa (lag 14 d, RR: 7.59, 95% CI: 4.45–12.95), respectively. The cumulative effects of PRS on the risk of HFMD among male children aged 4 ~ 6 years and rural populations were more significant. However, the cumulative risk effect of PRS on HFMD among children aged 4 ~ 6 years first decreased and then increased with the increase in lag days. At the same time, the cumulative risk effect of HFMD among children aged 0 ~ 3 years and ≥ 7 years continued to increase.

Low (4 hPa) and high (≥ 21 hPa) PRSDs were risk factors for HFMD, and the related peak existed at 24 hPa with a lag of 0 days (RR: 1.06, 95% CI: 0.77–1.45). With the increase in lag days, the cumulative risk effect of a low PRSD did not decrease significantly, while that of a high PRSD decreased rapidly. The cumulative effects of PRSD on the risk of HFMD among female children aged 4 ~ 6 years and urban populations were more significant. However, the cumulative risk effect of PRSD on HFMD in the ≥ 7-year-old population first decreased and then increased with the increase in lag days, and the RR rose to 17.12 after a lag of 14 days at 24 hPa.

Low (27–56%) and high (> 73%) RHU were risk factors for HFMD. With the increase in lag days, the cumulative effect of low RHU (< 35%) on the risk of HFMD increased rapidly (27%, lag 14 d, RR = 2.68, 95% CI: 1.44–4.99), while that of medium RHU (> 35%) decreased gradually, and that of RHU (41–56%) faded gradually. The cumulative effects of RHU on the risk of HFMD in female and rural populations were more significant.

Overall, the effect of PRE on HFMD was not significant, although high PRE (> 82 mm) had a significant effect on HFMD among males, children aged 4 ~ 6 years, and rural populations.

Low (≤ 3 °C) and high (> 21 °C) TEMs were risk factors for HFMD. With the increase in TEM and lag days, the cumulative effect of high TEM on the risk of HFMD increased rapidly (33 °C, lag 14 d, RR = 3.51, 95% CI: 2.84–4.34). The cumulative effects of TEM on the risk of HFMD among children aged 0 ~ 3 years and ≥ 7 years and rural populations were more significant. However, the risk of HFMD was not significantly different between men and women.

Low (< 7 °C) and high (> 12 °C) TEMDs were risk factors for HFMD. With the increase in TEMD and lag days, the cumulative effect of a low TEMD on the risk of HFMD decreased (1 °C, lag 3 d, RR = 1.27, 95% CI: 1.12–1.43), while the cumulative risk effect of a high TEMD continued to increase (17 °C, lag 14 d, RR = 2.04, 95% CI: 1.31–3.19). Compared with that observed in urban populations, the cumulative effect of TEMD on HFMD in rural populations was more prominent.

With the increase in WIN, its cumulative effect on the risk of HFMD first decreased and then increased. The RR value of the cumulative effect of WIN on the risk of HFMD lagging for 14 days decreased from 1.21 at 1 m/s to 0.93 at 3 m/s and then gradually increased rapidly to 955.45 at 9 m/s. The cumulative effects of WIN on the risk of HFMD among males, the ≥ 7-year-old population, and urban populations were more significant.

Low (2–4 h) SSD was a risk factor for HFMD, and the cumulative effect increased with increasing lag days (3 h, lag 14 d, RR = 1.06, 95% CI: 1.01–1.12).

**Fig. 6:**
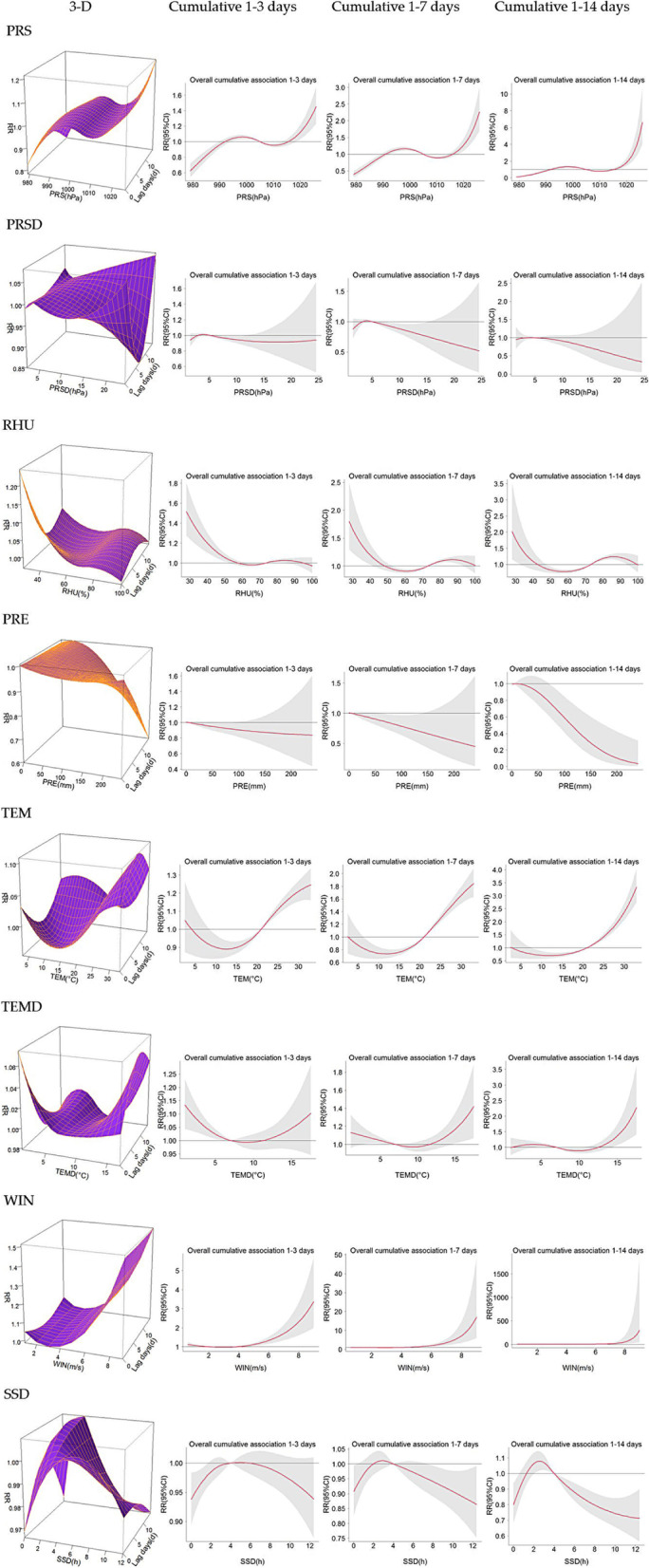
3-D plots and cumulative lag effect plots of the impacts of meteorological factors on the risk of HFMD

**Fig. 7:**
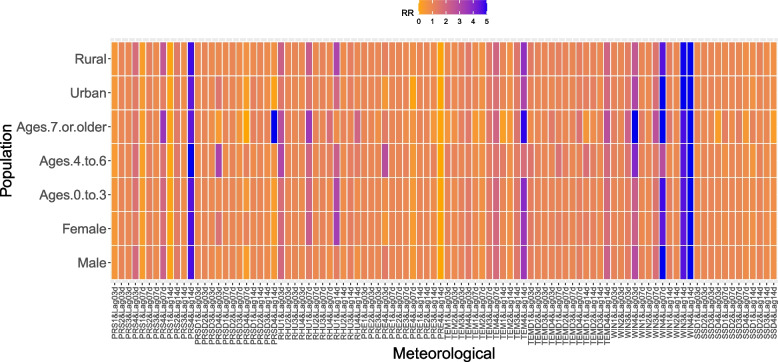
Cumulative effects of meteorological factors on the risk of HFMD in each population. (Note: **a** When RR > 5, it was counted as 5. **b** Meteorological values were divided into four grades, and the details are presented in Table 3. **c** The number of lag days was divided into three grades: 3 d, 7 d, and 14 d)

More meteorological characteristics related to HFMD are presented in Fig. 6, Fig. 7, and Table 3.

**Table 3:** Grading values of meteorological factors

Variables	1	2	3	4
PRS(hPa)	979	998	1010	1026
PRSD(hPa)	2	4	12	24
RHU(%)	27	65	83	100
PRE(mm)	1	3	50	244
TEM(°C)	3	13	25	33
TEMD(°C)	1	4	9	17
WIN(m/s)	1	3	6	9
SSD(h)	0	3	7	12

The values of different grades of meteorological factors for the cumulative effect analysis of population-stratified HFMD risk were set according to their minimum value, median value, mean value, maximum value, and the value when the pretested risk ratio (RR) was large

### LSTM forecast

**Fig. 8:**
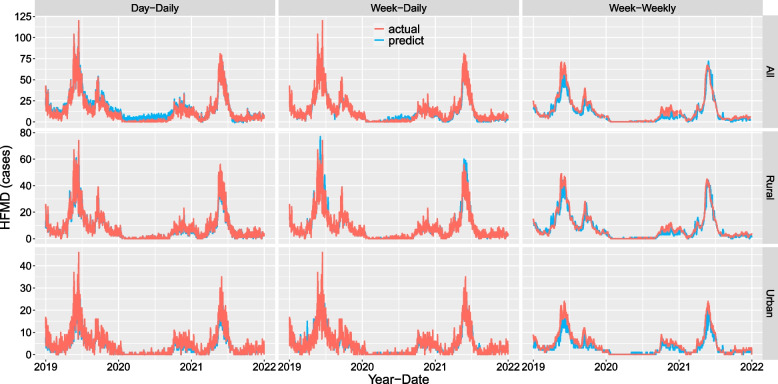
Predicted true HFMD values from 2019 to 2021 based on LSTM. (Note: Day-Daily: The multifactor value of 1 day was input to predict the cases of HFMD on the next day. Week-Daily: The multifactor value of 7 days was input to predict the cases of HFMD on the next day. Week-Weekly: The multifactor value of 7 days was input to predict the daily average cases of HFMD in the next 7 days)

**Fig. 9:**
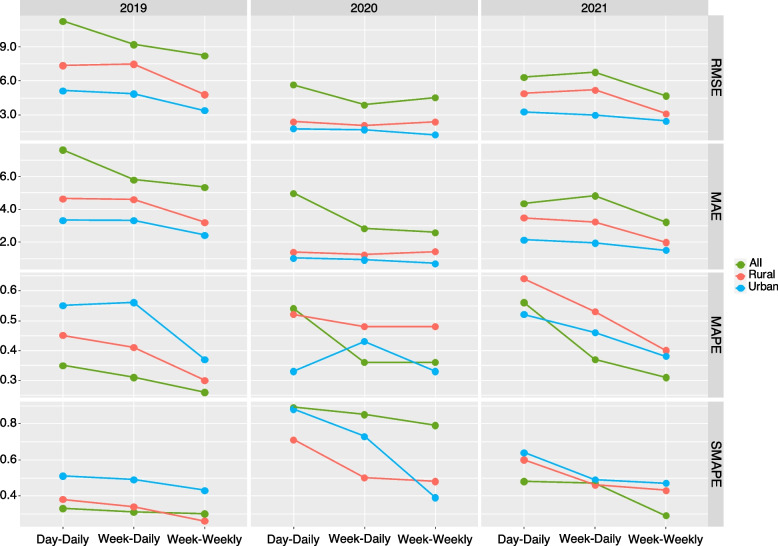
Evaluation indicators based on LSTM prediction

All, rural and urban HFMD cases were predicted and evaluated respectively. Figure 8 shows that the cases of HFMD predicted by the three methods from 2019 to 2021 were in good agreement with the actual values, and had high accuracy. Figure 9 shows that the RMSE, MAE, MAPE and SMAPE of using the weekly multifactor data to predict the cases of HFMD on the next day, from 2019 to 2021, were lower than those of using the daily multifactor data to predict the cases of HFMD on the next day. In particular, the RMSE, MAE, MAPE and SMAPE of using weekly multifactor data to predict the next week's daily average cases of HFMD were much lower, and similar results were also found in rural and urban areas, which indicating that this approach was more accurate.

## Discussion

Figure 4 shows that the time series fluctuations of HFMD and PRE had obvious consistency. Nevertheless, the DLNM showed that, overall, the effect of PRE on the risk of HFMD was not significant. However, high PRE (> 82 mm) significantly affected HFMD risk among male children aged 4 ~ 6 years and in rural populations. These results seemed contradictory. However, through Fig. 4, we found that the number of cases of HFMD in the peak period of PRE from 2020 to 2021 were substantially fewer than those in previous years. With the emergence of the coronavirus disease 2019 (COVID-19) pandemic, protective and control measures such as restricted movement, reduced physical contact in public places, frequent hand washing, appropriate ventilation, and disinfection of public areas were initiated. All these measures carried out to prevent coronavirus transmission reduced the probability of contracting HFMD. More importantly, the suspension of classes because of the COVID-19 pandemic substantially reduced the number of HFMD outbreaks, especially in 2020. Due to the impact of the COVID-19 pandemic, the incidence of HFMD decreased abnormally for two consecutive years from 2020–2021, which may have affected the accuracy of the assessment of the cumulative effect of PRE on HFMD through the DLNM.

Figure 5 shows the results of Spearman grade correlation analysis, which showed that PRE was significantly positively correlated with the risk of HFMD. In contrast, the relationship between TEMD and HFMD risk was not apparent. Nevertheless, DLNM analysis showed that the effect of PRE on HFMD risk was not significant, while low (< 7 °C) and high (> 12 °C) TEMDs were risk factors for HFMD. The DLNM integrates nonlinear dependence and delay effects and considers the control of potential confounding factors, while Spearman rank correlation analysis lacks these functions, which means that using a DLNM to analyse the impact of meteorological factors on infectious diseases can obtain more practical and specific results.

In this study, there was no significant correlation between PRE and HFMD risk. This result was consistent with those of some research reports [32, 33] but inconsistent with other research reports [13, 34]. This may be due to regional heterogeneity or the abnormal reduction in HFMD cases caused by COVID-19 prevention and control measures in recent years. Numerous studies have also emphasized the importance of temporal and spatial heterogeneity in meteorological impacts on infectious diseases [27, 35, 36]. In addition, this may be caused by differences in the analysis model scheme and data type. For example, in this study, RHU was significantly associated with HFMD risk, consistent with the findings of several daily value-based research reports [18, 37, 38], while some monthly value-based research reports showed no significant correlation between them [39, 40]. However, our results showed that the characteristics and value range of RHU affecting HFMD risk were different from those reported in other studies; it was not that the higher the humidity was, the more significant the impact. Moreover, this study showed that lower relative humidity is also a risk factor for HFMD. HFMD in Fuzhou has two peak outbreak periods in summer and autumn every year. Fuzhou has a typical subtropical monsoon climate. It is dominated by sunny, hot, and high-temperature weather in summer, with ample rainfall and high humidity. In autumn, the sky is clear and clouds are scarce, with sufficient sunshine, reduced humidity, and appropriate temperatures. During humid days, the HFMD virus could easily attach to small particles in the air or to toy surfaces; therefore, sharing toys and other supplies among children might promote the spread of the disease [41, 42]. In summer, increased RHU is usually accompanied by heavy rainfall in Fuzhou, so outdoor public facilities are frequently washed by rainwater, which reduces the attachment of pathogens and reduces children's outdoor activities when RHU is high. Thus, high (> 73%) RHU was a risk factor for HFMD in Fuzhou, and the cumulative risk effect increased first and then decreased with increasing RHU. Therefore, high (> 73%) RHU may be mainly due to the high incidence of HFMD in summer, and low humidity may be mainly due to the high incidence of HFMD in autumn.

There are few reports on the impact of PRS on HFMD. However, we found that the impact of PRS on the risk of HFMD increased gradually in waves with increasing PRS. Medium PRS (993–1005 hPa) and high PRS (> 1015 hPa) were risk factors for HFMD. In principle, the influencing factors of PRS include temperature, altitude, and air movement. PRS decreases with increasing TEM and increases with decreasing TEM. Fuzhou is mainly characterized by severe cold winters and a subtropical climate in summer and autumn. PRS increases the density of harmful gas and viruses floating in the air, allowing them to fall on the ground or objects quickly. For example, as one of the main pollutants, nitrogen dioxide (NO_2_) increases the risk of HFMD by affecting immunity, resulting in inflammation and weakening the body's resistance to viral infection [37]. The peak of PRS in Fuzhou is distributed in winter. However, this peak is accompanied by low temperatures (the average temperature in winter is 11 °C), and low temperatures are not conducive to the growth and transmission of the HFMD virus in the external environment. Therefore, the incidence of HFMD in winter is not high. In addition, the cumulative effect of PRS on HFMD risk increased with the increase in lag days, showing that the impact did not easily subside, which may play a chronic role.

This study showed that with the increase in TEM, the cumulative impact of high TEM (> 21 °C) on the risk of HFMD increases rapidly. Several studies have shown that the incidence of HFMD significantly increases as the temperature increases [33, 40, 43-46]. High temperatures can increase enterovirus growth and interfere with the inactivation and recovery of enteroviruses [47, 48]. Temperature can also affect the behavioural patterns of the host population; for instance, warm weather may encourage children to go out to public entertainment areas more often, thereby increasing their frequency of contact with each other and leading to more exposure to pathogens [49, 50]. In addition, the hands easily sweat in high temperatures, which is conducive to the breeding and cross-infection of viruses when in contact with the public. Children are even more active and sweat easily. However, in this study, we found that a low (≤ 3 °C) TEM was also a risk factor for HFMD. The possible underlying mechanism of HFMD can be explained by interactions of pathogens, host population structure, and environmental factors [34, 51, 52]. When the temperature is low, interactions often occur in relatively closed public places with poor ventilation and among people with poor handwashing habits. Moreover, in Fuzhou, RHU is usually very high in low-temperature seasons, such as the end of winter and early spring (not caused by heavy precipitation), which is conducive to the breeding and transmission of the virus.

The impact of the PRSD and TEMD on HFMD has not been reported, but in this study, we found that their low and high values were risk factors for HFMD. The cumulative effect of PRSD on the risk of HFMD among females was more significant, showing that immunity among females may be more susceptible to changes in air pressure. We also found that, compared with urban populations, the cumulative effect of TEMD on HFMD risk in rural populations was more prominent. Many rural areas in Fuzhou are distributed in mountainous regions, with apparent diurnal body temperature differences. In contrast, the differences in the TEMD and diurnal body temperature in urban areas are negligible due to the heat island effect. Nevertheless, the meteorological data measured in this study were from the same meteorological station. In other words, the meteorological values of urban and rural areas came from one meteorological station, and the measured values were the same, but the difference between the two meteorological environments was obvious, which may affect the meteorological evaluation of the HFMD risk effect.

To predict the incidence of HFMD more accurately through meteorological factors, we studied and evaluated various prediction methods. The methods commonly used in the prediction, such as the susceptible-infectious-recovery (SIR) model, autoregressive integrated moving average (ARIMA) model, and the recurrent neural network (RNN), have exhibited good performance, but they are still not satisfactory for the following reasons. The SIR model cannot fully use the information in the multidimensional input data; the ARIMA requires time series data to be stable after differential differentiation and can only capture linear relationships, not nonlinear relationships. At the same time, gradient extinction easily occurs in RNNs, and the problem of long-distance dependence cannot be handled [22-24, 29, 53].

LSTM is an advanced RNN with the ability to learn time patterns and store valuable memories longer [3]. Due to its unique design structure, LSTM can solve gradient extinction problems and nonlinear relationships. In addition, it can incorporate meteorological factors and is also suitable for predicting important events with very long intervals and delays in time series. It has been reported that the accuracy of using LSTM model to predict HFMD was better than other models [28, 54].

This study showed that the RMSE, MAE, MAPE and SMAPE values of the cases of HFMD predicted using the Day-Daily, Week-Daily, and Week-Weekly methods were low. This indicates that it was more accurate to predict HFMD cases using weekly multifactor data, especially to predict the daily average cases in the next week. The prediction of rural and urban areas also presented a similar situation, which further supports this result. Moreover, it was more in line with the actual work to predict the daily average cases of HFMD in the next week by using weekly multifactor data. At the same time, this also indicates that the meteorological indicators in this study can accurately predict the incidence of HFMD through LSTM models.

However, overfitting should be avoided during modelling. LSTM models involve the risk of underfitting or overfitting, which often results in poor prediction performance [28, 55]. In addition, the model's performance deteriorates when the number of memory neurons is less than 32 or the number of training rounds is less than 250 [28].

In summary, we introduced more abundant meteorological factors and screened out the common meteorological factor PRE in this study, which makes the multifactor parameter setting more comprehensive and reasonable. We also built a multifactor and multistep LSTM prediction model for infectious disease prevention that can flexibly adapt to the input parameters in different scenarios. In this study, combined with the common prediction methods of infectious disease prevention and control, the LSTM model was adapted to the input of the three prediction methods, and the incidence of HFMD cases in Fuzhou achieved accurate prediction results. We also recognize that using weekly multifactor data to predict HFMD cases, especially the daily average cases in the next week, is most accurate. Of course, according to the different needs of practical work, daily forecasts and weekly forecasts can be combined. These meteorological factors and prediction models can be incorporated into the HFMD early warning and prediction system of Fuzhou city and Fujian Province to provide a reference for formulating prevention strategies. They can also be used as risk predictions for adjusting people's lifestyles.

However, this study also has some limitations. First, although meteorological factors are very important for the spread of HFMD, social behaviours, the economy, population mobility and air quality may also affect the occurrence and spread of HFMD. Especially when comparing regions, such as urban and rural regions, the spread of infection is affected by differences in personal hygiene, including hand-washing, toileting habits, food handling habits and food handling personnel, although the prediction of urban and rural regions in this study is very accurate. Therefore, it may be more accurate to include more relevant influencing factors to predict HFMD. However, the influence of these factors can be reflected in the number of cases of HFMD to a certain extent. Therefore, when using HFMD and meteorological factors as multiple factors, it is necessary to regularly incorporate the latest HFMD and meteorological data into the revised prediction model within a short period of time and then repredict HFMD cases; half a year or one year may be appropriate. Second, in the past two years, COVID-19 prevention and control measures, such as the suspension of classes and reduction in outdoor activities, have substantially reduced the incidence of HFMD; thus, the impact of meteorology on HFMD and prediction research may have been affected, and the degree of impact needs to be further studied and evaluated. Third, the pathogenic stratification analysis of HFMD was not carried out in this study because most cases were clinically diagnosed and lacked laboratory results. Because the HFMD cases used in this study were reported by medical and health institutions, whereas laboratory test cases were scarce, the use of cases with laboratory test results for meteorological impact assessment would cause bias in the analysis results. Fourth, the topography and vertical structure are complex in Fuzhou; therefore, the meteorological conditions have also changed greatly. However, the meteorological data in this study came from one station, while the HFMD cases came from various medical and health institutions in the city, which may have affected the research results. Therefore, more meteorological station data need to be included in future studies.

## Conclusion

Meteorological factors such as PRS, PRSD, RHU, TEM, TEMD, WIN, and SSD significantly impact HFMD risk in Fuzhou. LSTM models combined with the meteorological factors in this study can accurately predict the risk of HFMD. It is more accurate to predict HFMD cases using weekly multifactor data, especially to predict the daily average cases in the next week. These meteorological factors and prediction models can be incorporated into an early warning and prediction system for HFMD in Fuzhou city and Fujian Province and could be used as a reference in other regions.

## Data Availability

The datasets that support the findings of this study are available from Fujian Provincial Centre of Disease Control and Prevention, Fujian Climate Center and meteorological data network of the China Meteorological Administration, but restrictions apply to the availability of these data, which were used under license for the current study, and so are not publicly available. Data are however available from the authors upon reasonable request and with permission of these three institutions (E-mail: hszhu33@126.com).

## Abbreviations

AIC: Akaike information criterion
ARIMA: Autoregressive integrated moving average
CI: Confidence interval
COVID-19: Corona virus disease 2019
DLNM: Distribution lag nonlinear models
GAMM: Models, generalized additive mixed model
GLM: Generalized linear model
LSTM: Long short-term memory
MAE: Mean absolute error
MAPE: Mean absolute percentage error
NO_2_: Nitrogen dioxide
PRE: Precipitation
PRS: Pressure
PRSD: Pressure difference
RHU: Relative humidity
RMSE: Root mean squared error
RNN: Recurrent neural network
RR: Risk ratio
SIR: Susceptible infectious recovery
SMAPE: Symmetric mean absolute percentage error
TEM: Temperature
TEMD: Temperature difference
SSD: Sunshine duration
WIN: Wind speed
ZINB: Spatiotemporal zero-inflated negative binomial
